# Detection of arrhythmia using an implantable cardiac monitor following a cryptogenic stroke: a single-center observational study

**DOI:** 10.1186/s40001-020-00424-3

**Published:** 2020-06-29

**Authors:** Alper Öner, Thomas Lips, Uwe Walter, Alexander Storch, Hüseyin Ince, Evren Caglayan, Seyrani Yücel, Jasmin Ortak, Christian Schmidt

**Affiliations:** 1 Department of Cardiology, Heart Center Rostock, University Hospital Rostock, Rostock, Germany; 2 Department of Neurology, University Hospital of Rostock, Rostock, Germany; 3 University Hospital of Rostock, Rostock, Germany; 4 Medizinische Klinik I im Zentrum für Innere Medizin (ZIM), Ernst-Heydemann-Str. 6, 18057 Rostock, Germany

**Keywords:** Atrial fibrillation, Cryptogenic shock, Transient ischemic attack, Insertable cardiac monitoring

## Abstract

**Background:**

Detection of atrial fibrillation (AF) after cryptogenic stroke (CS) has therapeutic implications, but the most effective type and optimal duration of monitoring have still to be defined. This study that involved patients with CS or transient ischemic attack (TIA), all of whom carried an implantable cardiac monitor (ICM), sought to assess the incidence of AF and other arrhythmia detected using tele-monitoring or interval-based follow-up by an internal cardiologist at the university medical center of Rostock (UMR) or an external cardiologist.

**Methods:**

The ICM implantation was performed during the inpatient stay in the neurology department, with inclusion and exclusion criteria jointly determined by the neurology and cardiology departments. Cardiologists programmed individual threshold values during ICM implantation, which were designed to instantly trigger an episode being recording and an alarm message being sent out. Outpatient care consisted of tele-monitoring of implants or interval-based follow-up care.

**Results:**

The indication for ICM implantation was made for 102 patients, 88 of whom underwent ICM implantation, with full documentation available for these 88 study patients. Within a median observation period of 21.5 months, AF occurred in 19 patients, with a median observation time to the event of 7 months. In all cases, AF detection was followed by immediate medical intervention. Comparing patients with and without AF revealed that the median age of the AF group exceeded by 10 years that of the other patients. Stroke recurrence was recorded in five patients, with a median observation time to the event of 9 months. Comparing patients with and without stroke recurrence revealed that the median age in the stroke recurrence group tended to be higher by 14 years. No statistically significant between-group differences were found with regard to integration into tele-monitoring, nor were there any differences identified between outpatient care at the UMR or in the outpatient sector.

**Conclusions:**

This study confirmed the feasibility of using an interdisciplinary and intersectoral therapeutic approach for monitoring CS patients with implanted ICMs. Further randomized studies are warranted to confirm these encouraging data. An open discussion concerning optimal care forms and opportunities for introducing digitizing care pathways appears warranted.

## Background

Implantable cardiac monitors (ICMs) are currently employed to detect, monitor, and record the heart rhythms of patients at risk of arrhythmia, including tachyarrhythmia, bradyarrhythmia, and atrial fibrillation (AF) [1]. The prevention of AF-related strokes is increasingly recognized as a global priority for public health. Although AF-related strokes are common and often associated with devastating consequences, they are largely preventable using anticoagulation therapy [2, 3]. Nevertheless, as AF can be asymptomatic and discontinuous, this silent risk factor may easily evade detection. Hence, the detection of AF following a cryptogenic stroke (CS) is a key indication for an ICM. In the literature, CS is defined as a stroke without any identifiable cause, even after extensive workup.

Recently, the EMBRACE or CRYSTAL-AF studies have revealed the ICM’s diagnostic sensitivity to detect numerous arrhythmias, especially AF in CS patients, with detection rates nearing 25% [4, 5]. In a meta-analysis conducted by Burkowitz et al. ICMs were proven superior in terms of diagnostic detection efficacy, compared to standard monitoring in the CS setting [6]. Moreover, this meta-analysis demonstrated that ICM monitoring of CS patients was associated with fewer stroke recurrences and increased quality-adjusted life expectancy, compared to standard care. Interestingly, this meta-analysis also demonstrated a decrease in CS-related costs for ICM patients [6]. Guidelines on AF management from The European Society of Cardiology (ESC) currently recommend long-term ICM implementation, especially in older ischemic stroke patients [7]. It is also important to note that manufacturers have recently offered direct information transfer via a smartphone, without a manufacturer-specific transmitter.

## Study objectives

The current study is embedded within the publicly funded project titled “Development and Evaluation of a Telemedical Competence Center for Individualized Therapy Management of Chronic Heart Disease Patients with Special Consideration of Patients with Cryptogenic Stroke through Networked Structures in Mecklenburg-Vorpommern”. This project necessitated digitization of the entire patient pathway, as well as a detailed description of the interdisciplinary and intersectoral therapeutic management of CS patients using ICM monitoring. With this in mind, our study objectives were fourfold: to investigate the incidence of detected events like stroke and AF; to examine the therapeutic equivalence of ICM follow-up using tele-monitoring compared to interval-based check-ups; to compare outcomes from the University Medical Center Rostock (UMR) follow-up and outpatient follow-up; to develop an organizational model for an e-health competence center.

## Methods

### Study design

The indication for ICM implantation for this single-center study was made during an inpatient stay in the neurology department, with inclusion and exclusion criteria jointly determined by the cardiology and neurology departments. A stroke was defined as CS if no clear etiology could be established despite the following extensive tests: full neurological assessments, including CHA_2_DS_2_-VASc score, brain computed tomography (CT) scan, or magnetic resonance imaging (MRI); CT-angiography of the head and neck; duplex ultrasonography of extra- and intracranial brain supplying arteries, resting electrocardiogram (ECG), 24-h Holter-ECG, and 72-h ECG-monitoring on the stroke unit; transthoracic or transesophageal echocardiography; screening for thrombophilic states or vasculitis, and standard laboratory analyses.

### Patient population

Eligible patients were those admitted to the neurology department of the UMR that had undergone ICM implantation following the occurrence of CS. The ICM had to be implanted during an inpatient stay, because outpatient implantation is not part of standard care. From November 1, 2017 onwards, applications were submitted to health-insurance companies for case-by-case decisions concerning outpatient implantation (off-label use). Patient inclusion started on February 3, 2016 and ended on October 31, 2018.

### ICM devices

Implant choice was at the attendant physician’s discretion, considering product availability. The ICM continuously detected the patient’s heart rhythm and automatically recorded events like AF or atrial tachyarrhythmia, ventricular fibrillation or tachyarrhythmia, bradyarrhythmia, and asystole. Cardiologists programmed individual threshold values during ICM implantation, which were designed to instantly trigger an episode being recording and an alarm message being sent out, where necessary. In this study, a distinction was made between AF and other arrhythmias.

### Outpatient care

The first follow-up was scheduled for 3 months after ICM implantation. During this visit, the patient was informed about the possibility of undergoing either implant tele-monitoring or interval-based follow-up, either by a resident cardiologist at the UMR or an external cardiologist. Patients were thus allowed to choose their preferred mode of follow-up.

Patients without tele-monitoring attended cardiac rhythm consultations at regular intervals. The events that were recorded and stored in the implant were telemetrically read using an external sensor on the implant. All anomalies were analyzed and documented by a cardiological expert to discern their clinical relevance. For patients that were not followed up at the UMR, a formal questionnaire was sent to the respective cardiologist to be filled.

**Fig. 1:**
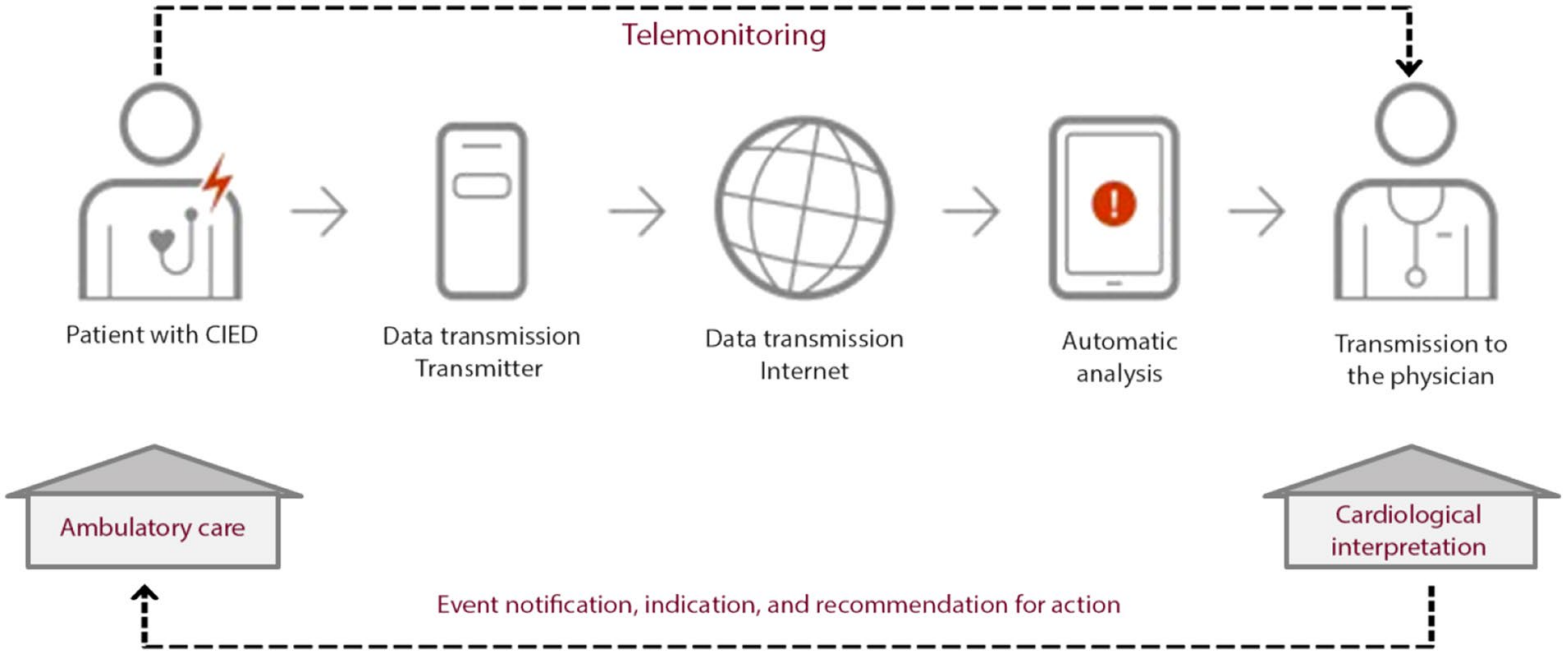
Workflow of active cardiac implants according to Biotronik group of companies

For patients undergoing tele-monitoring, daily information about parameters was automatically sent to the patient’s mobile device. Data stored in the implant were transmitted at night via the frequency band of the Medical Implant Communication Service (MICS). The system generated an alarm message for values outside the normal programmed range, reports of which were viewed daily by a specially trained nurse. However, clinically relevant events were documented by attending cardiologists and communicated to the patient’s external practitioner. Such communication was considered an intervention. Figure 1 further illustrates the structural workflow.

### Statistical analysis

Continuous variables were expressed as mean ± standard deviation (SD), and categorical variables were expressed as numbers (percentages). The normal distribution of samples was checked using the Kolmogorov–Smirnov test. In the case of normal distribution, between-group comparisons were made using the Student’s *t* test. In the absence of normal distribution, the Mann–Whitney U test was applied. Kaplan–Meier methodology was employed to determine the probability of stroke recurrence, AF, and other arrhythmias. Data for patients without an event were censored at the end of the observation period. Censored data constituted data pertaining to patients without any event during the observation period, whose ICM was explanted without any detected event, or those that were lost to follow-up or could not be observed during the study period. The level of significance was set at *p* < 0.05. All analyses were performed using IBM SPSS Statistics V22.0 for Windows.

### Ethical standards

The study was performed according to the 1964 principles of the Declaration of Helsinki and its later amendments. The project was approved by the Ethics Committee of the University Hospital Rostock on September 19, 2017. All patients provided their written informed consent concerning the use of their data for research purposes.

## Results

### Study population

The indication for ICM implantation was made for 102 patients, 88 of whom received an ICM implant. The remaining 14, who did not receive an ICM implant, were excluded from further investigation. The socio-demographic characteristics of both groups are summarized in Table 1. No significant differences in socio-demographic features were found between the two patient groups.

**Table 1:** Comparison of socio-demographic characteristics between patients with and without implant

Characteristics	Patients with implant *n* = 88	Patients without implant *n* = 14	*P*-value
Age (years), median (range)	66.5 (30–89)	68.5 (47–82)	0.556
Gender, *n* (%)			0.214
Male	54 (61.4)	11 (78.6)	
Female	34 (38.6)	3 (21.4)	
Event, *n* (%)			0.240
Stroke	80 (90.9)	14 (100)	
Transient ischemic attack	8 (9.1)	0	
Body mass index (kg/m^2^), median (range)	27 (19–49)	28 (20–34)	0.995
Comorbidity, *n* (%)			
Hypertension	59 (67.0)	10 (71.4)	0.745
Diabetes mellitus	20 (22.7)	2 (14.3)	0.476
Vasculopathy	19 (21.6)	1 (7.1)	0.206
Heart failure	3 (3.4)	0	0.483
CHA_2_DS_2_-VASc score, median (range)	4 (2–7)	4.5 (2–7)	0.870
Score 2, *n* (%)	7 (8.0)	2 (14.3)	
Score 3, *n* (%)	20 (22.7)	3 (21.4)	
Score 4, *n* (%)	19 (21.6)	2 (14.3)	
Score 5, *n* (%)	22 (25.0)	2 (14.3)	
Score 6, *n* (%)	11 (12.5)	3 (21.4)	
Score 7, *n* (%)	9 (10.2)	2 (14.3)	
Extrasystole, *n* (%)	64 (72.7)	0	
Number of drugs, median (range)	4 (1–14)	5 (2–10)	0.709
Smocking status, n (%)			0.069
Nonsmoker	49 (55.7)	8 (57.1)	
Smoker	25 (28.4)	1 (7.1)	
Ex-smoker	12 (13.6)	3 (21.4)	
No indication	2 (2.3)	2 (14.3)	

**Fig. 2:**
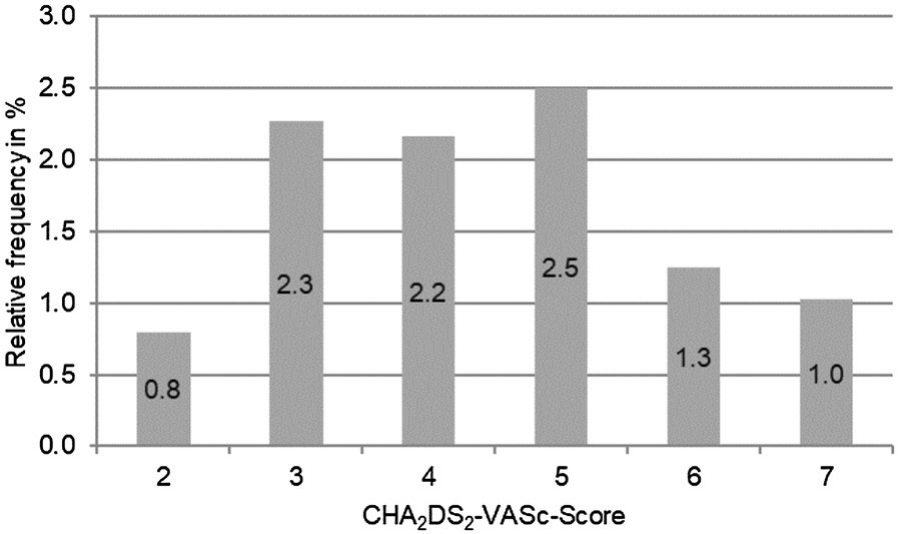
CHA_2_DS_2_-VASc distribution across the study population

Overall, 88 patients underwent ICM implantation following either a CS or transient ischemic accident (TIA), all of whom had complete data and event reports. Their median age was 66.5 years, and 54 were male and 34 female. All patients had experienced a thromboembolic event, consisting of a stroke for 80 patients and TIA for eight. The most common comorbidity was arterial hypertension, which was observed in 59 patients, followed by diabetes mellitus and vasculopathy in 20 and 19 patients, respectively. The patents’ median CHA_2_DS_2_-VASc score was 4 (range: 2–7), and its distribution across the study population is depicted in Fig. 2.

**Fig. 3:**
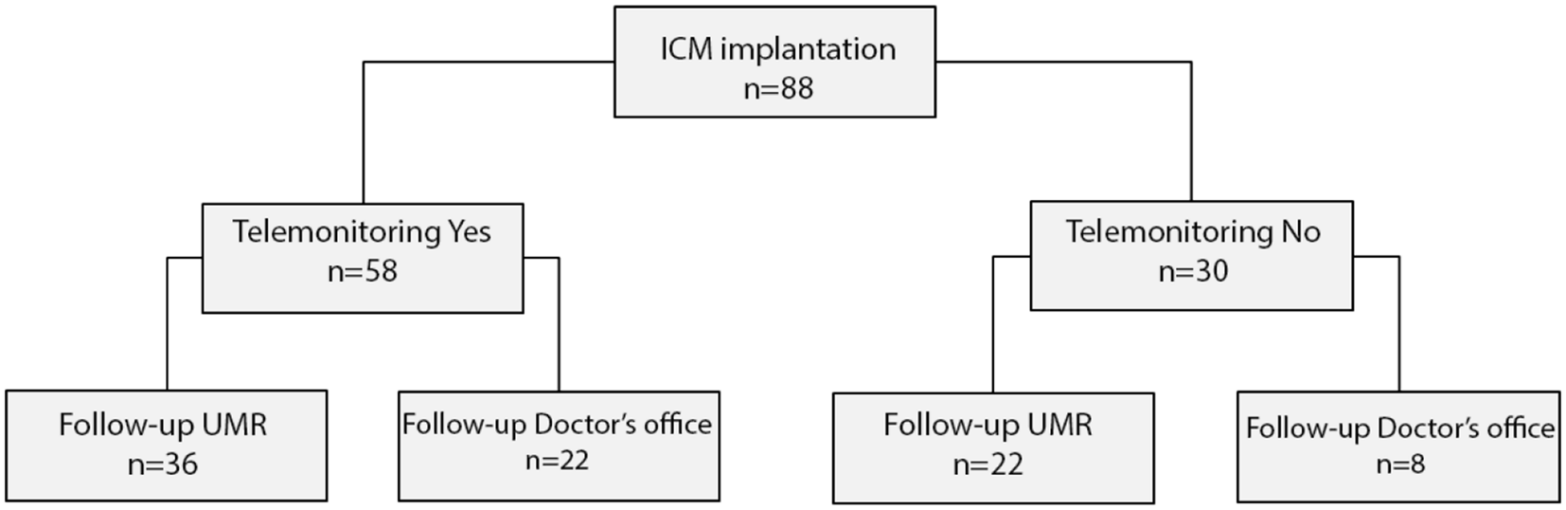
Flowchart of patient distribution

Table 2 provides information on implant characteristics and patient distribution depending by integration into tele-monitoring and aftercare type. With regard to ICMs, 58 devices were from Biotronik, 17 from Medtronic, and 13 from St. Jude. Tele-monitoring was activated in 58 (65.9%) patients. Of the 88 implanted patients, 58 were followed up at the UMR, compared to 30 in the outpatient sector (Fig. 3). For these latter patients, event reports were recorded retrospectively and made available for evaluation. For two of these patients, only data until the first aftercare visit could be collected, after which the patients were lost to follow-up.

**Table 2:** Implant characteristics, patient distribution, and ambulatory follow-up care

Parameter	Overall population (*n* = 88)
Implant manufacturer, *n* (%)	
Biotronik	58 (65.9)
Medtronic	17 (19.3)
St. Jude	13 (14.8)
Implant designation, *n* (%)	
Biomonitor2-AF	58 (65.9)
Reveal LINQ	2 (2.3)
Reveal XT	15 (17.0)
SJM confirm	13 (14.8)
Tele-monitoring, *n* (%)	
On	58 (65.9)
Off	30 (34.1)
Ambulatory follow-up care, *n* (%)	
Rostock University Medical Center	58 (65.9)
Doctor’s office	30 (34.1)
Observation period (months), median (range)	21.5 (1–33)

### AF events

**Fig. 4:**
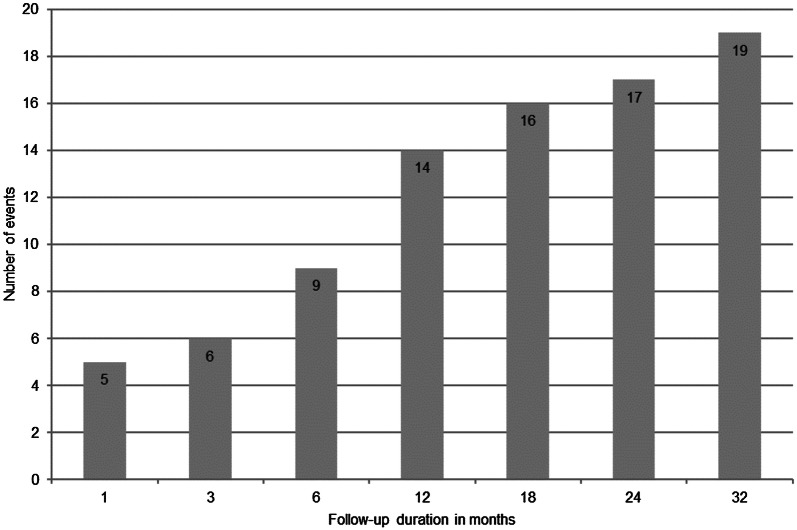
Cumulative occurrence of atrial fibrillation in the study group

During a median observation period of 21.5 months (range: 1–33), 19 AF events occurred. The cumulative order of these events is illustrated in Fig. 4. In these 19 cases, AF detection resulted in immediate intervention, and oral anticoagulation was initiated in 16/19 patients.

Based on the Kaplan–Meier methodology, the probability of patients not experiencing an AF event was 89.2% and 67.8% after 6 and 33 months, respectively. The median observation time to the first AF event was 7 months (range: 1–32).

Testing for differences between patients with AF and those without revealed that the AF group’s median age tended to be higher by 10 years compared to the AF-free group. However, statistical significance was not reached (Table 3). No other differences were identified.

**Table 3:** Comparison of demographic and pathological characteristics between patients with and without atrial fibrillation events

Parameter	No atrial fibrillation *n* = 69	Atrial fibrillation *n* = 19	*P*-value
Age (years), median (range)	64 (30–89)	74 (47–83)	0.060
Gender, *n* (%)			0.377
Male	44 (63.8)	10 (52.6)	
Female	25 (36.2)	9 (47.4)	
Event, *n* (%)			0.512
Stroke	62 (89.9)	18 (94.7)	
Transient ischemic attack	7 (10.1)	1 (5.3)	
Body mass index (kg/m^2^), median (range)	27 (19–49)	28 (23–44)	0.478
Comorbidity, *n* (%)			
Hypertension	47 (68.1)	12 (63.2)	0.684
Diabetes mellitus	17 (24.6)	3 (15.8)	0.415
Vasculopathy	16 (23.2)	3 (15.8)	0.488
Heart failure	3 (4.3)	0	0.355
CHA_2_DS_2_-VASc score, median (range)	4 (2–7)	5 (2–7)	0.346
Score 2, *n* (%)	6 (8.7)	1 (5.3)	
Score 3, *n* (%)	16 (23.2)	4 (21.1)	
Score 4, *n* (%)	16 (23.2)	3 (15.8)	
Score 5, *n* (%)	17 (24.6)	5 (26.3)	
Score 6, *n* (%)	7 (10.1)	4 (21.1)	
Score 7, *n* (%)	7 (10.1)	2 (10.5)	
Extrasystole, *n* (%)	52 (75.4)	12 (63.2)	0.290
Number of drugs, median (range)	4 (1–14)	5 (2–8)	0.992
Smocking status, *n* (%)			0.720
Nonsmoker	37 (53.6)	12 (63.2)	
Smoker	21 (30.4)	4 (21.1)	
Ex-smoker	9 (13.0)	3 (15.8)	
No indication	2 (2.9)	0	
Observation period (months), median (range)	23 (1–33)	7 (1–32)	*0.002*
Recurrent stroke during observation period, *n* (%)	XX	XX	*XX*

Significant *P*-value is in itlaics

### Other detected arrhythmias

In addition to AF, other arrhythmias documented as clinically relevant were detected in 19 patients. The median observation time to the first arrhythmia event was 7 months (range: 1–32). In 8 of these 19 patients, arrhythmia detection resulted in immediate intervention, which consisted of pacemaker implantation in three and medication adjustment in five patients. No significant differences were established between patients with another arrhythmia and those without.

Overall, 69 patients were censored: two that were lost to follow-up, six whose ICMs were explanted without any detection of AF, and 61 without another arrhythmia.

Overall, 38 clinically relevant arrhythmias were detected, 35 (92.1%) of which were observed within 18 months. An AF event and another arrhythmia were detected in combination in six patients, whereas 13 patients exhibited either intermittent AF or another arrhythmia.

### Stroke recurrence

Stroke recurrence was recorded in five patients, with a median observation time to stroke recurrence of 9 months in the relapse group.

Testing for differences between the populations with and without stroke recurrence revealed that the median age in the stroke recurrence group was 80 years, compared to 66 in the no-recurrence group. This difference, however, only tended toward statistical significance (*p* = 0.068), and no other relevant difference was observed.

### Aftercare concepts

For outpatient follow-up care, continuous implant tele-monitoring was employed in 58 patients and interval-based implant control was used in the remaining 30. The median observation time for tele-medically treated patients was 20.5 months (range: 1–33) versus 29 months (range: 1–33) for the other group. These two strategies were carried out at both the UMR and external cardiological practices. No statistically significant differences were found between the groups with and without tele-monitoring, nor were any differences identified between aftercare at the UMR and external practices.

These different aftercare populations were compared in order to identify any differences in AF detection rates. In the patient group that was followed up at the UMR, the AF detection rate was 24.1% (*n* = 14) compared to 16.7% (*n* = 5) in external practices. With regard to tele-monitoring, 15.5% of AF events were detected in the tele-monitoring-positive group (*n* = 9) versus 33.3% in the tele-monitoring**-**negative group. No relevant differences were detected with respect to the implants used.

## Discussion

The relevance of cardiac monitoring for AF in the aftermath of a CS has been well documented. Patient monitoring has revealed that a number of CS survivors display AF episodes, thus making it essential to properly detect these AF events. This ensures patients benefit from secondary prevention, which primarily consists of anticoagulant therapy. The association between AF and CS has also been strengthened by ICM-based intensive cardiac monitoring, since it has been proven that continuous ECG monitoring of CS patients using ICM is significantly superior, in terms of AF detection, compared to intermittent, interval-based monitoring strategies, such as 24-h, 48-h, and 7-day Holter recordings [8, 9]. Although several randomized studies have meanwhile demonstrated the relevance of cardiac monitoring using ICM devices, only a few real-life studies exist that involved a follow-up period exceeding 12 months. Our study is thus among the first real-life cohorts of CS patients, and its median follow-up is 21.5 months.

### AF detection rate

In most studies involving CS patients, AF detection rates were categorized at 1, 6, and 12 months. In our study, AF detection rates at these time intervals were 5.7%, 10.2%, and 15.9%, respectively, while the AF detection rate for the entire observation period was 21.6%. These findings align with other published reports. In the CRYSTAL-AF trial, which involved 221 implant patients, detection rates were 8.9% at 6 months, and 12.4% at 12 months [4]. Similar to our study, Ziegler et al. examined AF detection rates based on ICM monitoring at 1 and 6 months in a real-world study that involved 1247 patients [10]. The corresponding figures were 4.6% at 1 month and 12.20% at 6 months. In their conclusions, the authors stated that their study results entirely aligned with the CRYSTAL-AF. Moreover, Milstein et al. followed up a total of 343 CS patients by means of ICM [1]. These authors reported detection rates of 5.0% at 1 month and 21.0% at 1 year. In brief, our cohort study revealed AF detection rates that roughly align with these published figures.

### CHA_2_DS_2_-VASc score

In our study population, the median CHA_2_DS_2_-VASc score was 4 (range: 2–7), while the median CHADS_2_ score was 3 (range: 2–5). Here, again, our findings align with data in major published reports. The CRYSTAL-AF study [4] and Milstein et al. report [1] both revealed CHADS_2_ score percentage distributions that were quite similar to those observed in our patient cohort. It should be noted that both scores are deemed suitable for identifying patients at high risk of AF and thromboembolic complications. However, the CHA_2_DS_2_-VASc score is more sensitive than the CHADS_2_ score, in terms of predicting thromboembolic events. Examining our CS patient cohort, however, failed to reveal any statistically significant links between the CHA_2_DS_2_-VASc score and the patient groups that had and did not have an arrhythmia event or stroke recurrence. Nevertheless, it must be stressed that the median score for the population that exhibited an arrhythmia event or stroke recurrence was one value higher than that of the event-free population. Unlike our study, the SURPRISE study succeeded in demonstrating a statistically significant link between the CHA_2_DS_2_-VASc score and AF events. Essentially, this last study revealed that a higher CHA_2_DS_2_-VASc score was linked to a higher likelihood of AF or another arrhythmia event occurring [11].

### Stroke recurrence

Five stroke recurrences were registered in our CS cohort, and all recurrences were observed within a post-implantation period of 16 months. While an association between stroke recurrence and AF events could not be established, stroke recurrence tended to be linked to older age. No other associations were identified. Remarkably, all stroke recurrences were observed in male patients. As the stroke recurrence rate in our CS cohort was significantly lower than the rates reported in other studies, our 5.4% stroke recurrence rate compares favorably with the figures of the SURPRISE study, which reported a 14.98% recurrence rate (stroke + TIA) over a mean 569-day observation [11]. The low stroke recurrence rate that was registered in our cohort further reinforces the collective benefits that are derived from continuous arrhythmia detection and event-triggered interventions across the entire treatment path. However, additional randomized studies are now needed to investigate the evidence that a combination of continuous implant monitoring and event-based therapy initiation is associated with a drastic reduction in stroke recurrences. In a 2019 review paper, which was published in the Eur Heart J and titled “Screening for Atrial Fibrillation: a Call for Evidence”, Jones et al. highlighted several gaps in the evidence and reasoning for and against different screening strategies [12].

### Event-triggered interventions

Our investigation initially sought to demonstrate the superiority of continuous tele-monitoring, in terms of AF detection rates, in comparison with interval-based follow-up, in addition to initiating appropriate treatment as soon as possible. As a matter of fact, the detection of an arrhythmia can only impact a patient’s outcome if it is promptly followed by interventional strategies, which mainly consist of anticoagulant therapy. Appropriately, all AF events that occurred in our CS cohort were promptly followed by an immediate intervention, and anticoagulation therapy was prescribed in 84% of patients. These data perfectly align with the study results of Bötsch et al., which was conducted in a similar setting [13]. These authors reported anticoagulant therapy initiation recorded in 85% of patients upon AF detection. In the setting of our cohort study, the patients who were prescribed anticoagulant therapy were not further followed-up. Further studies should been designed and implemented to better describe these patients’ long-term outcome on anticoagulant therapy. With respect to the other clinically relevant arrhythmias detected in our CS cohort, their rate was 43.2%, along with an intervention rate of 30.1%. It is noteworthy that these arrhythmia events, as well as the corresponding interventions, were all recorded within 18 months of the stroke event.

### Paradoxical finding

A paradoxical finding occurred in the course of our study: the AF detection rate of interval-based follow-up was higher than that of tele-monitoring (33.3% versus 13.0%). This appears to contradict the outcome of previous studies using similar designs and settings [4, 5]. This inconsistency is difficult to explain. One feature that may be worth noting is that the median observation time between both patient populations differed by 8.5 months, in favor of the interval-based follow-up cohort. A number of questions are, however, left unanswered. It is still questionable whether all patients referred from the Neurology to the Cardiology Departments underwent comparable measurements and met similar inclusion criteria. Moreover, information pertaining to patients directly transferred to the outpatient sector was not fully available, thereby precluding the full assessment of the comparability of the two groups.

### Study perspectives

This is the first study conducted in the Mecklenburg/Vorpommern area that employed such an interdisciplinary and intersectoral therapeutic approach for secondary prevention in the post-stroke aftercare. Socio-demographic, clinical, and event data were collected all along the patient’s treatment path, and these were brought together from different clinical and outpatient areas, using a multidisciplinary approach. The prerequisites were proven to provide anyone involved in the process adequate access to information. The possibility of involving even more actively the patient in the therapeutic pathway, along with its impact on compliance and treatment adherence, should be further explored in longer-term studies.

### Study limitations

The results of this study should be interpreted in light of several methodological limitations, the major one being its non-randomized study design, which may have biased patient selection. Keeping this major limitation in mind, it is essential that our study results be further confirmed in larger-sized randomized comparative trials. The CS patients most likely to benefit from tele-monitoring must also be better defined, in addition to an in-depth cost–benefit analysis.

## Conclusions

This study has clearly confirmed the feasibility of using an interdisciplinary and intersectoral therapeutic approach for CS patients with implanted ICMs. This study, which has so far been unique in the Mecklenburg/Vorpommern area, could be the starting point for a comprehensive assessment of current care in stroke patients in Mecklenburg/Vorpommern area. An open discussion concerning optimal care forms and opportunities for introducing digitizing treatment paths appears warranted.

## Data Availability

The datasets used and/or analyzed during the present study are available from the corresponding author on reasonable request.

## Abbreviations

AF: Atrial fibrillation
ICM: Implantable cardiac monitor
TIA: Transient ischemic attack
